# Supplementary material for: An in vivo translation-reporter system for the study of protein synthesis in zebrafish embryos
Source: Biol Open. 2018 Dec 15;7(12):bio039362. doi: 10.1242/bio.039362 (PMC6310896; doi:10.1242/bio.039362)
Supplement: Supplementary information [file biolopen-7-039362-s1.pdf]

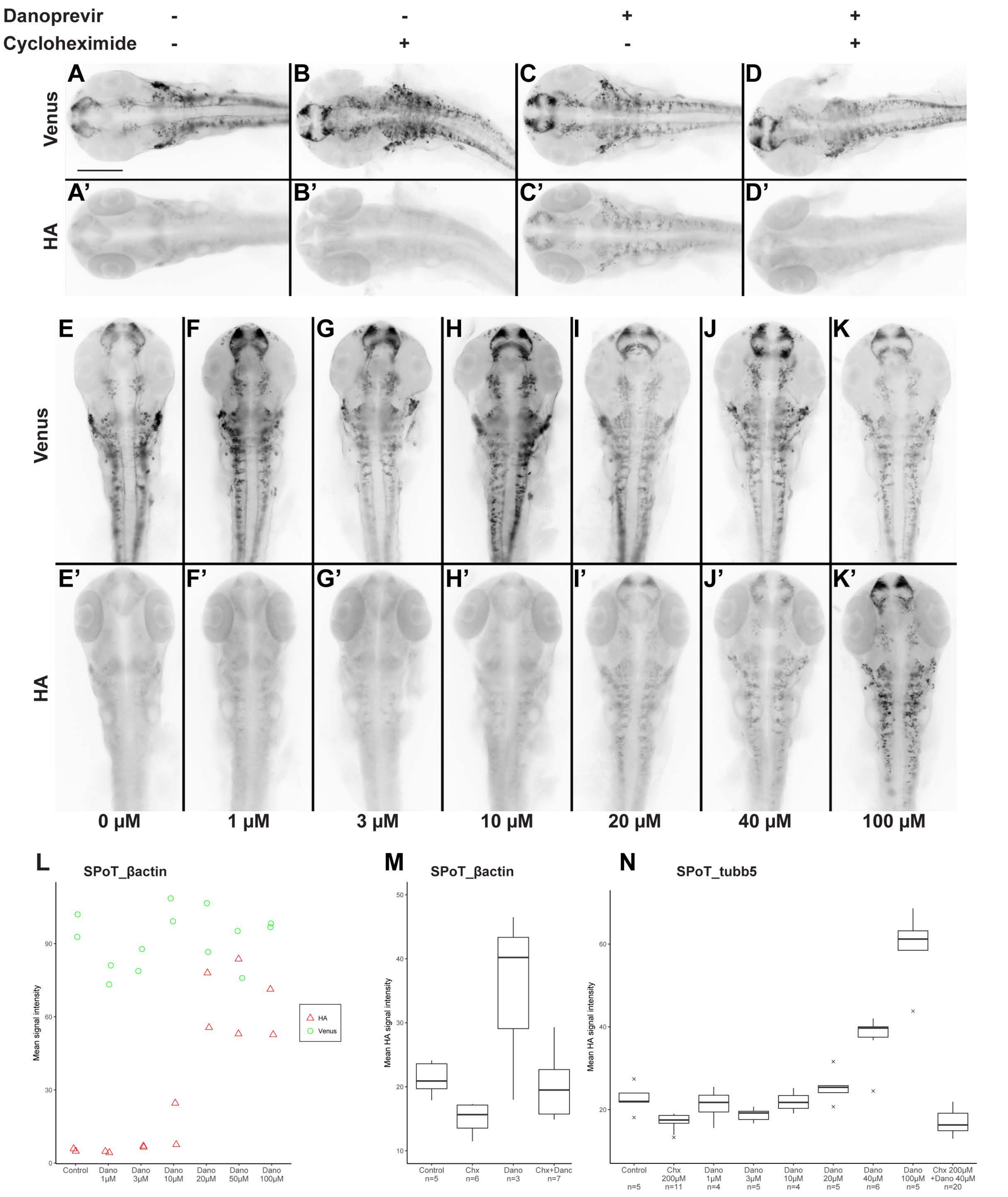

Biology Open • Supplementary information

Figure S1. Validation of the SPoT method

(A-D') Flatmounted immunostaining for Venus (top row) and HA (bottom row) of Tg(SPoT_tubb5) embryos incubated for 1h30 with Danoprevir (C,C'), Cycloheximide (B,B'), both (D,D') or none (A,A'). **(E-K')** Flatmounted immunostaining for Venus (top row) and HA (bottom row) of Tg(SPoT_tubb5) embryos incubated for 1h30 with increasing concentrations of Danoprevir.
Scale bar: 400 µm.
(L) Graphical representation of mean fluorescence values of Venus (green circles) and HA (red triangles) immunostaining signals in Tg(SPoT_chicken ß-actin) embryos treated for 1h30 with each indicated concentration of Danoprevir, including embryos pictured in Fig.1F-L'.
(M) Graphical representation of mean fluorescence value of HA immunostaining signals in Tg(SPoT_chicken ß-actin) embryos treated for 1h with each indicated condition, including embryos pictured in Fig.1B-E'.
(N) Graphical representation of mean fluorescence value of HA immunostaining signals in Tg(SPoT_ tubb5) embryos treated for 1h30 with each indicated condition, including embryos pictured in (A-K').
Each boxplot represents median values and limits of 1st and 3rd quartile.

Biology Open • Supplementary information

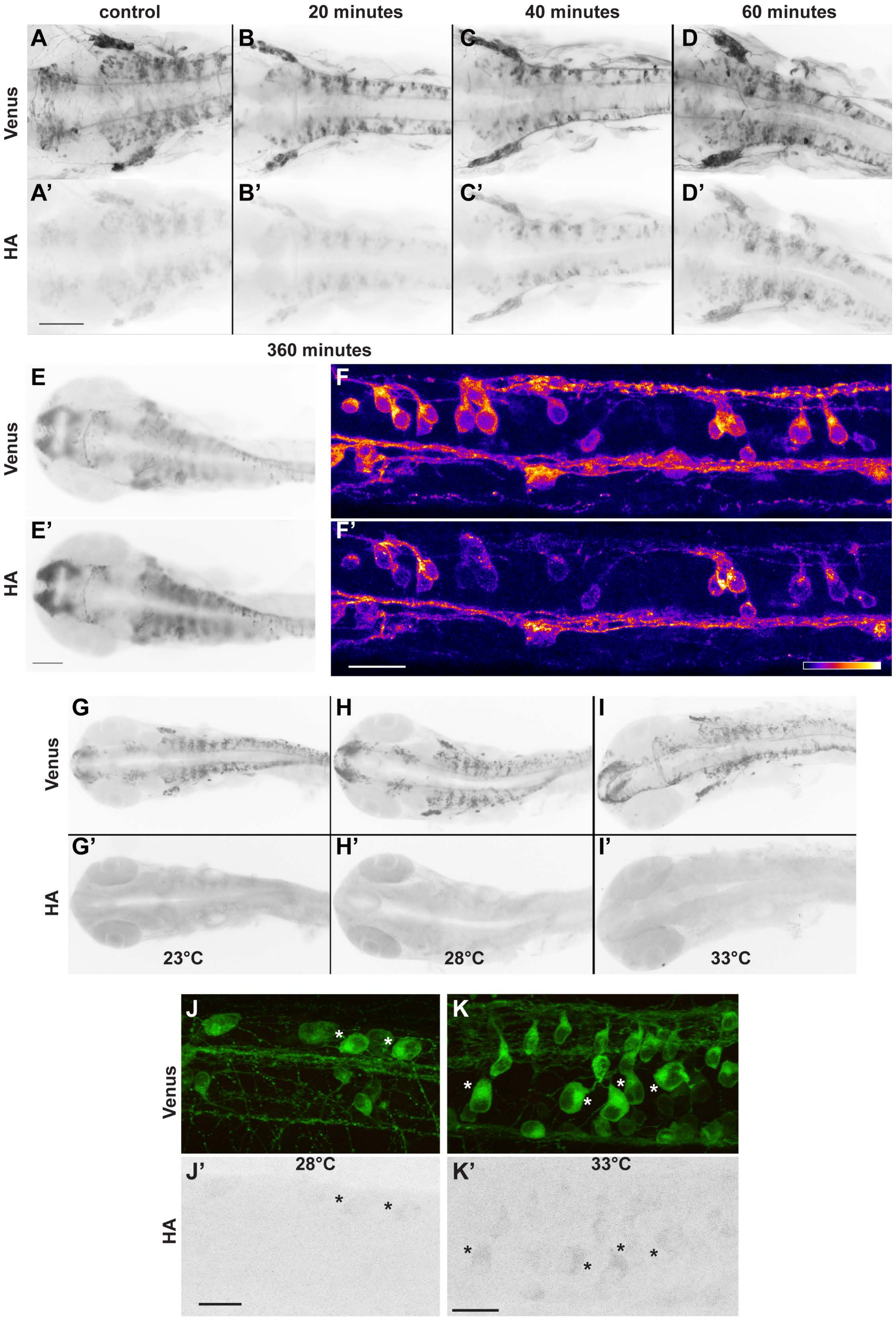

Biology Open • Supplementary information

Figure S2: Time and temperature calibration of the SPoT method

(A-F') Time course of HA accumulation following addition of 40 µm Danoprevir protease inhibitor.
Flatmounted immunostaining for Venus (top row) and HA (bottom row) of Tg(SPoT_chicken ß-actin) embryos incubated with Danoprevir for 0 (A,A'), 20 (B,B'), 40 (C,C'), 60 (D,D'), or 360 (E-F') minutes.
(G-K') Effect of temperature on residual HA immunoreactivity in untreated transgenic embryos
Flatmounted immunostaining for Venus (top row) and HA (bottom row) of Tg(SPoT_chicken ß-actin) embryos raised at 23°C (G,G'), 28°C (H,H',J,J') or 33°C (I,I',K,K') for at least 4 hours before fixation.
Asterisks in (J-K') highlight cells where HA-labelled (=unprocessed SPoT protein) is detected between the nucleus and axon initial segment before drug treatment, indicating that this pattern, although largely amplified by Danoprevir treatment, is not artificially generated by the drug itself.
Scale bars: 200 µm in (A-E'). 20 µm in (F-F', J-K').

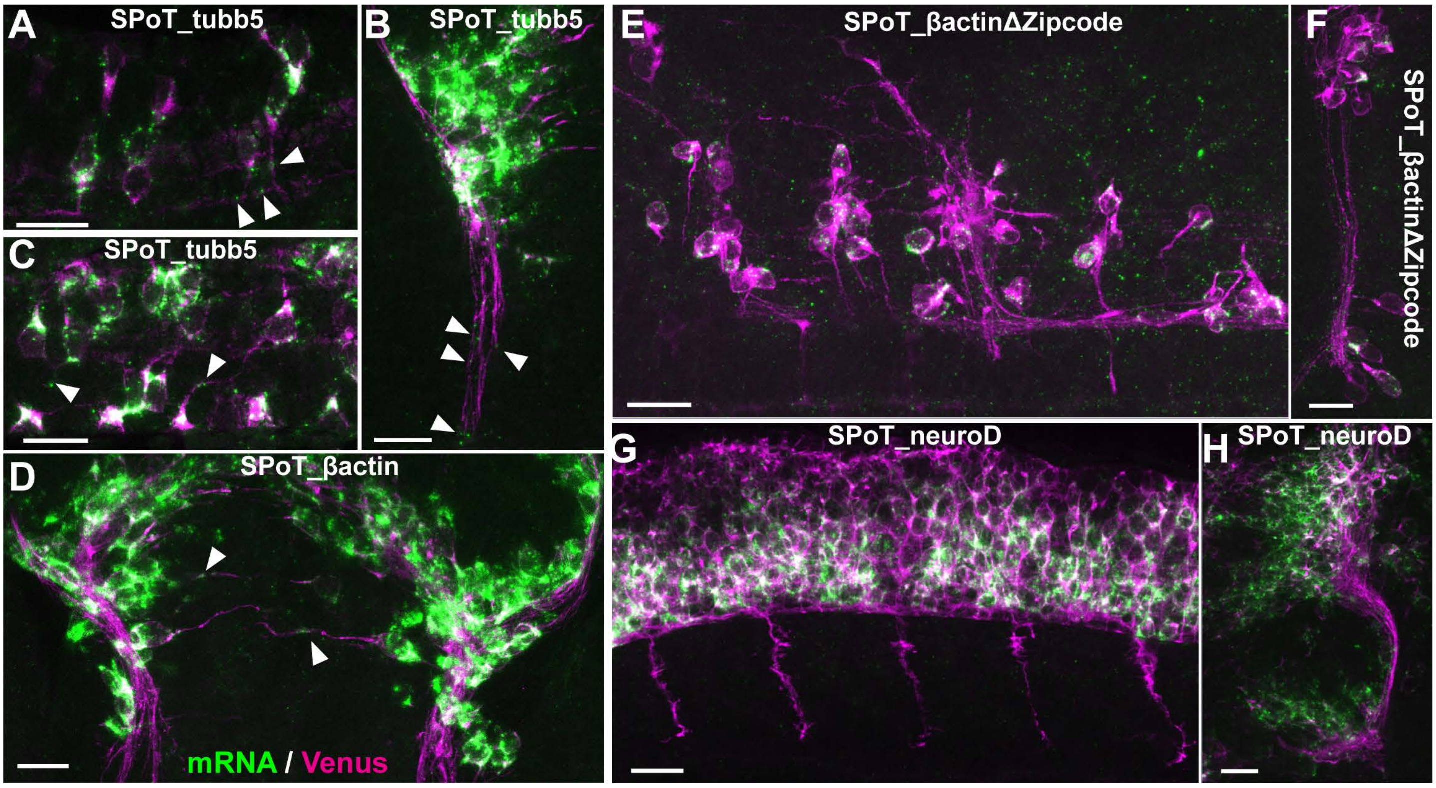

Figure S3. Localisation of the SPoT reporter transcripts

Representative examples of mRNA localisation patterns of SPoT transgenes. Cyan: Venus mRNA in situ hybridization. Magenta: Venus immunochemistry, labelling the SPoT-expressing neurons.
(A-C) Transgenic line = Tg(SPoT_tubb5)
(D) Tg(SPoT_chicken ß-actin)
(E-F) Tg(SPoT_chicken ß-actin-ΔZipcode)
(G-H) Tg(SPoT_neuroD)
White arrowheads indicate spots of in situ hybridization signal in neurites.
Scale bars: 20 µm.
